# Numerical Solution of Some Types of Fractional Optimal Control Problems

**DOI:** 10.1155/2013/306237

**Published:** 2013-12-09

**Authors:** Nasser Hassan Sweilam, Tamer Mostafa Al-Ajami, Ronald H. W. Hoppe

**Affiliations:** ^1^Department of Mathematics, Faculty of Science, Cairo University, Giza 12613, Egypt; ^2^Institute of Mathematics, University of Augsburg, 86159 Augsburg, Germany; ^3^Department of Mathematics, University of Houston, Houston, TX 77204-3008, USA

## Abstract

We present two different approaches for the numerical solution of fractional optimal control problems (FOCPs) based on a spectral method using Chebyshev polynomials. The fractional derivative is described in the Caputo sense. The first approach follows the paradigm “optimize first, then discretize” and relies on the approximation of the necessary optimality conditions in terms of the associated Hamiltonian. In the second approach, the state equation is discretized first using the Clenshaw and Curtis scheme for the numerical integration of nonsingular functions followed by the Rayleigh-Ritz method to evaluate both the state and control variables. Two illustrative examples are included to demonstrate the validity and applicability of the suggested approaches.

## 1. Introduction

FOCP refers to the minimization of an objective functional subject to dynamical constraints on the state and the control which have fractional order models. Fractional order models are sometimes more appropriate than conventional integer order models to describe physical systems [1–4]. For example, it has been shown that materials with memory and hereditary effects and dynamical processes including gas diffusion and heat conduction in fractal porous media can be more adequately modeled by fractional order models [5]. Numerical methods for solving FOCPs have been suggested in [6–9].

This paper presents two numerical methods for solving some types of FOCPs where fractional derivatives are introduced in the Caputo sense. These numerical methods rely on the spectral method where Chebyshev polynomials are used to approximate the unknown functions. Chebyshev polynomials are widely used in numerical computation [10, 11].

For the first numerical method, we follow the approach “optimize first, then discretize” and derive the necessary optimality conditions in terms of the associated Hamiltonian. The necessary optimality conditions give rise to fractional boundary value problems that have left Caputo and right Riemann-Liouville fractional derivatives. We construct an approximation of the right Riemann-Liouville fractional derivatives and solve the fractional boundary value problems by the spectral method. The second method relies on the strategy “discretize first, then optimize.” The Clenshaw and Curtis scheme [12] is used for the discretization of the state equation and the objective functional. The Rayleigh-Ritz method provides the optimality conditions in the discrete regime.

The paper is organized as follows: in Section 2, some basic notations and preliminaries as well as properties of the shifted Chebyshev polynomials are introduced.  Section 3 contains the necessary optimality conditions of the FOCP model.  Section 4 is devoted to the approximations of the fractional derivatives. In Section 5, we develop two numerical schemes and present two illustrative examples to demonstrate the validity and applicability of the suggested approaches. Finally, in Section 6, we provide a brief conclusion and some final remarks.

## 2. Basic Notations and Preliminaries

### 2.1. Fractional Derivatives and Integrals

**Definition 1**

Let *x* : [*a*, *b*] → ℝ be a function, let *α* > 0 be a real number, and let *n* = ⌈*α*⌉, where ⌈*α*⌉ denotes the smallest integer greater than or equal to *α*. The left (left RLFI) and right (right RLFI) Riemann-Liouville fractional integrals are defined by

(1) Iatαx(t)=1Γ(α)∫at(t−τ)α−1x(τ)dτ (left  RLFI),Itbαx(t)=1Γ(α)∫tb(τ−t)α−1x(τ)dτ (right  RLFI).

The left (left RLFD) and right (right RLFD) Riemann-Liouville fractional derivatives are given according to

(2) Datαx(t) =1Γ(n−α)dndtn∫at(t−τ)n−α−1x(τ)dτ (left  RLFD),Dtbαx(t) =(−1)nΓ(n−α)dndtn∫tb(τ−t)n−α−1x(τ)dτ (right  RLFD).

Moreover, the left (left CFD) and right (right CFD) Caputo fractional derivatives are defined by means of

(3) DaCtαx(t) =1Γ(n−α)∫at(t−τ)n−α−1x(n)(τ)dτ (left  CFD),DtCbαx(t) =(−1)nΓ(n−α)∫tb(τ−t)n−α−1x(n)(τ)dτ (right  CFD).

The relation between the right RLFD and the right CFD is as follows [13]:

(4) DtCbαx(t)=Dtbαx(t)−∑k=0n−1x(k)(b)Γ(k−α+1)(b−t)k−α.

Further, it holds

(5) D0Ctαc=0, where  c  is  a  constant,D0Ctα  tn={0,for  n∈  ℕ0,  n<⌈α⌉Γ(n+1)Γ(n+1−α)tn−α,for  n∈  ℕ0,  n≥⌈α⌉,

where *ℕ*_0_ = {0,1, 2,…}. We recall that, for *α* ∈ *ℕ*, the Caputo differential operator coincides with the usual differential operator of integer order. For more details on the fractional derivatives definitions and their properties, we refer the reader to [3, 8, 14, 15].

### 2.2. Shifted Chebyshev Polynomials

The well-known Chebyshev polynomials are defined on the interval [−1,1] and can be determined by the following recurrence formula [16]:

(6) $$\begin{array}{c} T_{n+1}\left(z\right)=\;2zT_{n}\left(z\right)-T_{n-1}\left(z\right),\\ T_{0}\left(z\right)=1,\;T_{1}\left(z\right)=z,\;n=1,2,\ldots . \end{array}$$

The analytic form of the Chebyshev polynomials *T*_*n*_(*z*) of degree *n* is as follows:

(7) $$\begin{array}{c} T_{n}\left(z\right)=\overset{\left\lfloor n/2\right\rfloor }{\underset{i=0}{\sum }}{\left(-1\right)}^{i}2^{n-2i-1}\frac{n\left(n-i-1\right)!}{\left(i\right)!\left(n-2\;i\right)!}\;z^{n-2i}, \end{array}$$

where ⌊*n*⌋ denotes the biggest integer less than or equal to *n*. The orthogonality condition reads

(8) $$\begin{array}{c} \overset{1}{\underset{-1}{\int }}\frac{T_{i}\left(z\right)T_{j}\left(z\right)}{\sqrt{1-z^{2}}}dz=\{\begin{array}{cc} \pi , & \text{for}\;\;i=j=0;\\ \frac{\pi }{2}, & \text{for}\;\;i=j\neq \;0;\\ 0, & \text{for}\;\;i\neq \;j. \end{array} \end{array}$$

In order to use these polynomials on the interval [0, *L*], we use the so-called shifted Chebyshev polynomials by introducing the change of variable *z* = (2*t*/*L*) − 1. The shifted Chebyshev polynomials are defined according to

(9) $$\begin{array}{c} T_{n}^{*}\left(t\right)=T_{n}\left(\frac{2t}{L}-1\right),\\ \text{where}\;T_{0}^{*}\left(t\right)=1\;T_{1}^{*}\left(t\right)=\frac{2t}{L}-1. \end{array}$$

Their analytic form is given by

(10) Tn∗(t)=n∑k=0n(−1)n−k22k(n+k−1)!(2k)!(n−k)!Lktk,          n=1,2,….

We note that (10) implies that *T*_n_*(0) = (−1)^*n*^, *T*_n_*(*L*) = 1. Further, it is easy to see that the orthogonality condition reads

(11) $$\begin{array}{c} \overset{L}{\underset{0}{\int }}T_{j}^{*}\left(t\right)T_{k}^{*}\left(t\right)w\left(t\right)dt=\delta_{jk}h_{k}, \end{array}$$

with the weight function $w(t)=1/\sqrt{Lt-t^{2}}$,  *h*_*k*_ = (*b*_*k*_/2)*π*,  *b*_0_ = 2,  *b*_*k*_ = 1, for *k* ≥ 1.

A function *y* ∈ *L* ^2^([0, *L*]) can be expressed in terms of shifted Chebyshev polynomials as

(12) $$\begin{array}{c} y\left(t\right)=\overset{\infty }{\underset{j=0}{\sum }}c_{n}T_{n}^{*}\left(t\right), \end{array}$$

where the coefficients *c*_*n*_ are given by

(13) $$\begin{array}{c} c_{n}=\frac{1}{h_{n}}\overset{L}{\underset{0}{\int }}y\left(t\right)T_{n}^{*}\left(t\right)w\left(t\right)dt,\;n=0,1,\ldots . \end{array}$$

## 3. Necessary Optimality Conditions

Let *α* ∈ (0,1) and let *L*, *f* : [*a*, +*∞*[×ℝ^2^ → ℝ be two differentiable functions. We consider the following FOCP [8]:

(14a) $$\begin{array}{c} \text{minimize}\;J\left(x,u,T\right)=\overset{T}{\underset{a}{\int }}L\left(t,x\left(t\right),u\left(t\right)\right)dt, \end{array}$$

subject to the dynamical system

(14b) M1x˙(t)+M2DaCtαx(t)=f(t,x(t),u(t)),

(14c) $$\begin{array}{c} x\left(a\right)=x_{a},\;\;x\left(T\right)=x_{T}, \end{array}$$

where *M*_1_, *M*_2_ ≠ 0,  *T*,  *x*_*a*_, and *x*_*T*_ are fixed real numbers.

**Theorem 2 (see [8])**

If (*x*, *u*, *T*) is a minimizer of (14a)–(14c), then there exists an adjoint state *λ* for which the triple (*x*, *u*, *λ*) satisfies the optimality conditions

(15a) M1x˙(t)+M2DaCtαx(t)=∂H∂λ(t,x(t),u(t),λ(t)),

(15b) M1λ˙(t)−M2DtTαλ(t)=−∂H∂x(t,x(t),u(t),λ(t)),

(15c) $$\begin{array}{c} \frac{\partial H}{\partial u}\left(t,x\left(t\right),u\left(t\right),\lambda \left(t\right)\right)=0, \end{array}$$

for all *t* ∈ [*a*, *T*], where the Hamiltonian *H* is defined by

(16) $$\begin{array}{c} H\left(t,x,u,\lambda \right)=L\left(t,x,u\right)+\lambda f\left(t,x,u\right). \end{array}$$

**Remark 3**

Under some additional assumptions on the objective functional *L* and the right-hand side *f*, for example, convexity of *L* and linearity of *f* in *x* and *u*, the optimality conditions (15a)–(15c) are also sufficient.

## 4. Numerical Approximations

In this section, we provide numerical approximations of the left CFD and the right RLFD using Chebyshev polynomials. We choose the grid points to be the Chebyshev-Gauss-Lobatto points associated with the interval [0, *L*]; that is,

(17) $$\begin{array}{c} t_{r}=\frac{L}{2}-\frac{L}{2}\cos\left(\frac{\pi r}{N}\right),\;\;r=0,1,\ldots ,N. \end{array}$$

Clenshaw and Curtis [12] introduced an approximation *y*_*N*_ of the function *y*. We reformulate it to be used with respect to the shifted Chebyshev polynomials as follows:

(18) $$\begin{array}{c} y_{N}\left(t\right)={\overset{N}{\underset{n=0}{\sum }}}^{\prime \prime }a_{n}T_{n}^{*}\left(t\right),\;\;a_{n}=\frac{2}{N}{\overset{N}{\underset{r=0}{\sum }}}^{\prime \prime }y\left(t_{r}\right)T_{n}^{*}\left(t_{r}\right). \end{array}$$

Here, the summation symbol with double primes denotes a sum with both first and last terms halved.

### 4.1. Approximation of the Left CFD

In the sequel, some basic results for the approximation of the fractional derivative _0_^*C*^ *D*_*t*_^*α*^ *y*(*t*) are given.

**Theorem 4 (see [17])**

An approximation of the fractional derivative of order *α* in the Caputo sense of the function *y* at *t*_*s*_ is given by

(19) D0CtαyN(ts)≅∑r=0Ny(tr)ds,rα,   α>0,

where

(20) $$\begin{array}{c} d_{s,r}^{\alpha }=\frac{4\theta_{r}}{N}\overset{N}{\underset{n=\left\lceil \alpha \right\rceil }{\sum }}\;\overset{N}{\underset{j=0}{\sum }}\;\overset{n}{\underset{k=\left\lceil \alpha \right\rceil }{\sum }}\frac{n\theta_{n}}{b_{j}}\frac{{\left(-1\right)}^{n-k}\left(n+k-1\right)!\Gamma \left(k-\alpha +1/2\right)T_{n}^{*}\left(t_{r}\right)T_{j}^{*}\left(t_{s}\right)}{L^{\alpha }\Gamma \left(k+1/2\right)\left(n-k\right)!\Gamma \left(k-\alpha -j+1\right)\Gamma \left(k-\alpha +j+1\right)}, \end{array}$$

where  *s*, *r* = 0,1,…, *N*, with *θ*_0_ = *θ*_*N*_ = 1/2,  *θ*_*i*_ = 1 for all *i* = 1,2,…, *N* − 1.

An upper bound for the error in the approximation of the fractional derivative _0_^*C*^ *D*_*t*_^*α*^ of the function *y* is given as follows.

**Theorem 5 (see [18])**

Let _0_^*C*^ *D*_*t*_^*α*^ *y*_*N*_(*t*) be the approximation of the fractional derivative _0_^*C*^ *D*_*t*_^*α*^ of the function *y* as given by (19). Then, it holds that

(21) ||D0Ctαy(t)−D0CtαyN(t)||2 ≤∑n=0N′′anΩn(G(tk−α;T0∗,…,TN∗)G(T0∗,…,TN∗))1/2,

where

(22) Ωn=∑k=⌈α⌉n(((−1)n−k2n(n+k−1)!×Γ(k−α+12))×(bjLαΓ(k+12)(n−k)!Γ(k−α−j+1)×Γ(k−α+j+1))−1),G(x;y1,y2,…,yn) =|〈x,x〉〈x,y1〉⋯〈x,yn〉〈y1,x〉〈y1,y1〉⋯〈y1,yn〉⋮⋮⋱⋮〈yn,x〉〈yn,y1〉⋯〈yn,yn〉|.

### 4.2. Approximation of the Right RLFD

Let *f* be a sufficiently smooth function in [0, *b*] and let *J*(*s*; *f*) be defined as follows:

(23) $$\begin{array}{c} J\left(s;f\right)=\overset{b}{\underset{s}{\int }}{\left(t-s\right)}^{-\alpha }f^{\prime }\left(t\right)dt,\;0<s<b. \end{array}$$

From (3) and (4), we deduce that

(24) Dsbαf(s)=f(b)Γ(1−α)(b−s)−α+J(s;f)Γ(1−α).

We approximate *f*(*t*),  0 ≤ *t* ≤ *b*, by a sum of shifted Chebyshev polynomials *T*_*k*_(2*t*/*b* − 1) according to

(25) $$\begin{array}{c} f\left(t\right)\approx p_{N}\left(t\right)={\overset{N}{\underset{k=0}{\sum }}}^{\prime \prime }a_{k}T_{k}\left(\frac{2t}{b}-1\right),\\ a_{k}=\frac{2}{N}{\overset{N}{\underset{j=0}{\sum }}}^{\prime \prime }f\left(t_{j}\right)T_{k}\left(\frac{2t_{j}}{b}-1\right), \end{array}$$

where  *t*_*j*_ = (*b*/2) − (*b*/2)cos⁡(*πj*/*N*), *j* = 0,…, *N*, and obtain

(26) $$\begin{array}{c} J\left(s;f\right)\approx J\left(s;p_{N}\right)=\overset{b}{\underset{s}{\int }}p_{N}^{\prime }\left(t\right){\left(t-s\right)}^{-\alpha }dt. \end{array}$$

**Lemma 6**

Let *p*_*N*_ be the polynomial of degree *N* as given by (25). Then, there exists a polynomial *F*_*N*−1_ of degree *N* − 1 such that

(27) ∫sx[pN′(t)−pN′(s)](t−s)−αdt =[FN−1(x)−FN−1(s)](x−s)1−α.

**Proof**

Let *p*_*N*_′(*t*) − *p*_*N*_′(*s*) be expanded in a Taylor series at *t* = *s*:

(28) $$\begin{array}{c} p_{N}^{\prime }\left(t\right)-p_{N}^{\prime }\left(s\right)=\overset{N-1}{\underset{k=1}{\sum }}A_{k}\left(s\right){\left(t-s\right)}^{k}. \end{array}$$

Then,

(29) ∫sx[pN′(t)−pN′(s)](t−s)−αdt =  ∑k=1N−1Ak(s)∫sx(t−s)k−αdt =  [(t−s)1−α∑k=1N−1Ak(s)(t−s)kk−α+1]sx.

The assertion follows, if we choose

(30) $$\begin{array}{c} F_{N-1}\left(x\right)=\overset{N-1}{\underset{k=0}{\sum }}\frac{A_{k}\left(s\right){\left(x-s\right)}^{k}}{k-\alpha +1}, \end{array}$$

with an arbitrary constant *A*_0_(*s*).

In view of (27), we have

(31) J(s;pN)=∫sbpN′(t)(t−s)−αdt=[pN′(s)1−α+FN−1(b)−FN−1(s)](b−s)1−α.

Moreover, _*s*_ *D*_*b*_^*α*^ *f*(*s*) can be approximated by means of

(32) Dsbαf(s)≈f(b)Γ(1−α)(b−s)−α+J(s;pN)Γ(1−α).

We express *F*_*N*−1_(*t*) in (31) by a sum of Chebyshev polynomials and provide the recurrence relation satisfied by the Chebyshev coefficients. Differentiating both sides of (27) with respect to *x* yields

(33) {pN′(x)−pN′(s)}(x−s)−α =FN−1′(x)(x−s)1−α  +{FN−1(x)−FN−1(s)}(1−α)(x−s)−α,

whence

(34) pN′(x)−pN′(s) =FN−1′(x)(x−s)+{FN−1(x)−FN−1(s)}(1−α).

To evaluate *F*_*N*−1_(*s*) in (31), we expand *F*_*N*−1_′(*x*) in terms of the shifted Chebyshev polynomials as

(35) $$\begin{array}{c} F_{N-1}^{\prime }\left(x\right)={\overset{N-2}{\underset{k=0}{\sum }}}^{\prime }b_{k}T_{k}\left(\frac{2x}{b}-1\right),\;0\leq x\leq b, \end{array}$$

where the summation symbol with one prime denotes a sum with the first term halved. Integrating both sides of (35) gives

(36) FN−1(x)−FN−1(s) =b4∑k=1N−1bk−1−bk+1k{Tk(2xb−1)−Tk(2sb−1)},

where *b*_*N*−1_ = *b*_*N*_ = 0. On the other hand, we have

(37) $$\begin{array}{c} \left(x-s\right)F_{N-1}^{\prime }\left(x\right)=\frac{b}{2}F_{N-1}^{\prime }\left(x\right)\left\{\left(\frac{2x}{b}-1\right)-\left(\frac{2s}{b}-1\right)\right\}. \end{array}$$

By using the relation *T*_*k*+1_(*u*) + *T*_*k*−1_(*u*) = 2*uT*_*k*_(*u*) and (35), it follows that

(38) (x−s)FN−1′(x) =b4∑k=0N−1′{bk+1−2(2sb−1)bk+bk−1}Tk(2xb−1),

where *b*_−1_ = *b*_1_. Let

(39) $$\begin{array}{c} p_{N}^{\prime }\left(x\right)={\overset{N-1}{\underset{k=0}{\sum }}}^{\prime }c_{k}T_{k}\left(\frac{2x}{b}-1\right). \end{array}$$

Inserting *F*_*N*−1_(*x*) − *F*_*N*−1_(*s*) and (*x* − *s*)*F*_*N*−1_′(*x*) as given by (36) and (38) into (34) and taking (39) into account, we get

(40) {1−1−αk}bk+1−2(2sb−1)bk +{1+1−αk}bk−1=4bck,   1≤k.

The Chebyshev coefficients *c*_*k*_ of *p*_*N*_′(*x*) as given by (39) can be evaluated by integrating (39) and comparing it with (25):

(41) $$\begin{array}{c} c_{k-1}=c_{k+1}+\frac{4k}{b}a_{k},\;k=N,N-1,\ldots ,1, \end{array}$$

with starting values *c*_*N*_ = *c*_*N*+1_ = 0, where *a*_*k*_ are the Chebyshev coefficients of *p*_*N*_(*x*).

## 5. Numerical Results

In this section, we develop two algorithms (Algorithms A and B) for the numerical solution of FOCPs and apply them to two illustrative examples.

**Example 1**

We consider the following FOCP from [8]:

(42a) $$\begin{array}{c} \min\;J\left(x,u\right)=\overset{1}{\underset{0}{\int }}{\left(tu\left(t\right)-\left(\alpha +2\right)x\left(t\right)\right)}^{2}dt, \end{array}$$

subject to the dynamical system

(42b) x˙(t)+D0Ctαx(t)=u(t)+t2

and the boundary conditions

(42c) $$\begin{array}{c} x\left(0\right)=0,\;\;x\left(1\right)=\frac{2}{\Gamma \left(3+\alpha \right)}. \end{array}$$

The exact solution is given by

(43) $$\begin{array}{c} \left(\overset{-}{x}\left(t\right),\overset{-}{u}\left(t\right)\right)=\left(\frac{2t^{\alpha +2}}{\Gamma \left(\alpha +3\right)},\frac{2t^{\alpha +1}}{\Gamma \left(\alpha +2\right)}\right). \end{array}$$

*Algorithm A*. The first algorithm for the solution of (42a)–(42c) follows the “optimize first, then discretize” approach. It is based on the necessary optimality conditions from Theorem 2 and implements the following steps. 

*Step *1. Compute the Hamiltonian

(44) $$\begin{array}{c} H={\left(tu\left(t\right)-\left(\alpha +2\right)x\left(t\right)\right)}^{2}+\lambda \left(u\left(t\right)+t^{2}\right). \end{array}$$

*Step *2. Derive the necessary optimality conditions from Theorem 2:

(45a) $$\begin{array}{c} \dot{\lambda }\left(t\right)-{\;}_{t}D_{1}^{\alpha }\lambda \left(t\right)=-\frac{\partial H}{\partial x}=2\left(\alpha +2\right)\left(tu\left(t\right)-\left(\alpha +2\right)x\left(t\right)\right), \end{array}$$

(45b) $$\begin{array}{c} \dot{x}\left(t\right)+{\;}_{0}^{C}D_{t}^{\alpha }x\left(t\right)=\frac{\partial H}{\partial \lambda }=u\left(t\right)+t^{2}, \end{array}$$

(45c) $$\begin{array}{c} 0=\frac{\partial H}{\partial u}=2t\left(tu\left(t\right)-\left(\alpha +2\right)x\left(t\right)\right)+\lambda . \end{array}$$

Use (45c) in (45a) and (45b) to obtain

(46a) −λ˙(t)+Dt1αλ(t)=(α+2)tλ(t),

(46b) $$\begin{array}{c} \dot{x}\left(t\right)+{\;}_{0}^{C}D_{t}^{\alpha }x\left(t\right)=-\frac{\lambda }{2t^{2}}+\frac{\left(\alpha +2\right)}{t}x\left(t\right)+t^{2}. \end{array}$$

*Step *3. By using Chebyshev expansion, get an approximate solution of the coupled system (46a), (46b) under the boundary conditions (42c).

*Step *3.1. In order to solve (46a) by the Chebyshev expansion method, use (18) to approximate *λ*. A collocation scheme is defined by substituting (18), (19), and (32) into (46a) and evaluating the results at the shifted Gauss-Lobatto nodes *t*_*s*_, *s* = 1,2,…, *N* − 1. This gives

(47) −∑r=0Nds,r1λ(tr)+λ(1)Γ(1−α)(1−ts)−α +J(ts;pn)Γ(1−α)=α+2tsλ(ts),

*s* = 1,2,…, *N* − 1, where *d*_*s*,*r*_^1^ is defined in (20). The system (47) represents *N* − 1 algebraic equations which can be solved for the unknown coefficients *λ*(*t*_1_), *λ*(*t*_2_),…, *λ*(*t*_*N*−1_). Consequently, it remains to compute the two unknowns *λ*(*t*_0_),  *λ*(*t*_*N*_). This can be done by using any two points *t*_*a*_, *t*_*b*_∈]0,1[ which differ from the Gauss-Lobatto nodes and satisfy (46a). We end up with two equations in two unknowns:

(48) λ˙(ta)+Dt1αλ(ta)=α+2taλ(ta),λ˙(tb)+Dt1αλ(tb)=α+2tbλ(tb).

*Step *3.2. In order to solve (46b) by the Chebyshev expansion method, we use (18) to approximate *x*. A collocation scheme is defined by substituting (18), (19), and the computed *λ* into (46b) and evaluating the results at the shifted Gauss-Lobatto nodes *t*_*s*_, *s* = 1,2,…, *N* − 1. This results in

(49) ∑r=0Nds,r1x(tr)+∑r=0Nds,rαx(tr) =−λ(ts)2ts2+α+2tsx(ts)+ts2,   s=1,2,…,N−1,

where *d*_*s*,*r*_^1^ and *d*_*s*,*r*_^*α*^ are defined in (20). By using the boundary conditions, we have *x*(*t*_0_) = 0 and *x*(*t*_*N*_) = 2/Γ(3 + *α*). The system (49) represents *N* − 1 algebraic equations which can be solved for the unknown coefficients *x*(*t*_1_), *x*(*t*_2_),…, *x*(*t*_*N*−1_).

**Figure 1:**
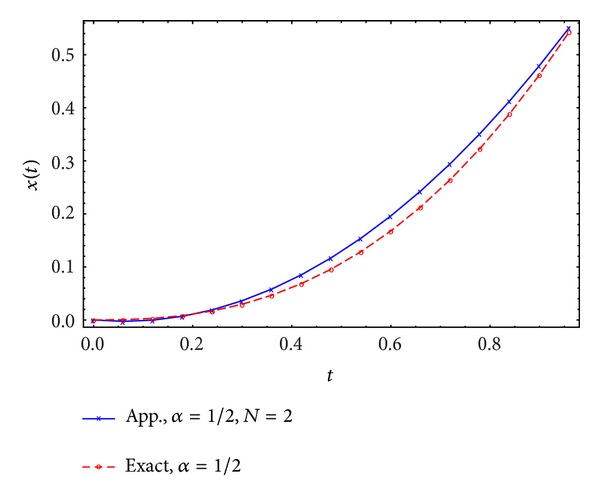
Exact and approximate state.

**Figure 2:**
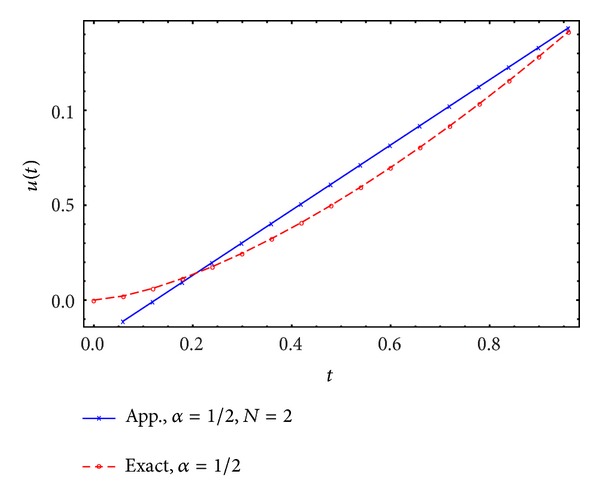
Exact and approximate control.

**Figure 3:**
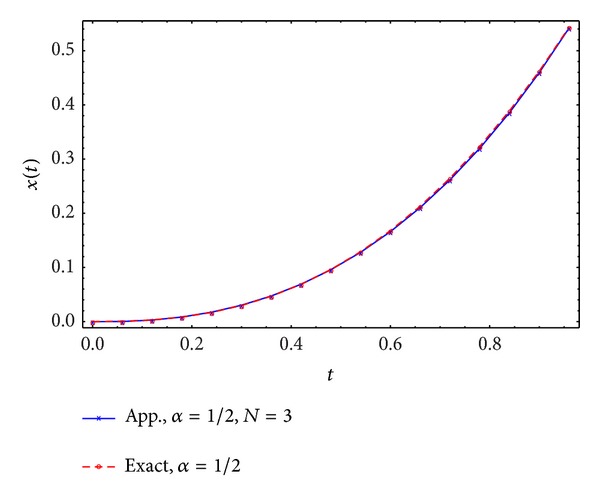
Exact and approximate state.

**Figure 4:**
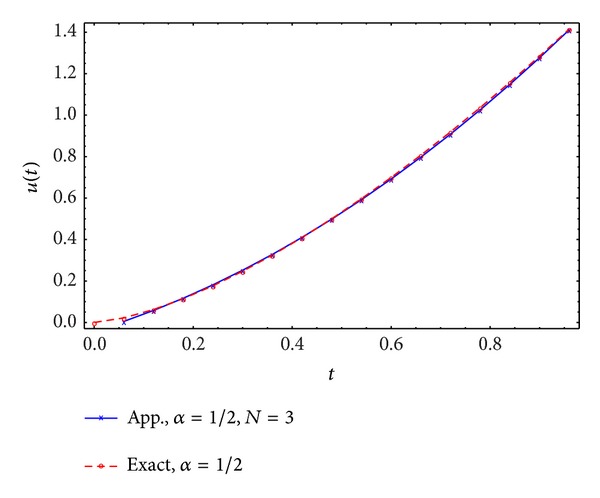
Exact and approximate control.

Figures 1, 2, 3, and 4 display the exact and approximate state *x* and the exact and approximate control *u* for *α* = 1/2 and *N* = 2,3.

Table 1 contains the maximum errors in the state *x* and in the control *u* for *N* = 2,  *N* = 3, and *N* = 5.

*Algorithm B*. The second algorithm follows the “discretize first, then optimize” approach and proceeds according to the following steps. 

*Step *1. Substitute (42b) into (42a) to obtain

(50) min⁡  J=∫01(t[x˙(t)+  0CDtαx(t)−t2]−(α+2)x(t))2dt.

*Step *2. Approximate *x* using the Clenshaw and Curtis formula (18) and approximate the Caputo fractional derivative _0_^*C*^ *D*_*t*_^*α*^ *x* and $\dot{x}$ using (19). Then, (50) takes the form

(51) min⁡  J=∫01(t[∑r=0Ndt,r1x(tr)+∑r=0Ndt,rαx(tr)−t2]−(α+2)∑n=0N′′anTn∗(t))2dt,

where *d*_*t*,*r*_^*α*^ is defined as in (20) replacing *t*_*s*_ by *t*. 

*Step *3. Use *t* = (1/2)(*η* + 1) to transform (51) to

(52) min⁡  J=  12∫−11(12(η+1)‍×[∑r=0Ndη,r1x(ηr)+∑r=0Ndη,rαx(ηr)−(12(η+1))2]−(α+2)∑n=0N′′anTn∗(η))2dη.

*Step *4. Use the Clenshaw and Curtis formula [12]

(53) $$\begin{array}{c} \overset{1}{\underset{-1}{\int }}F\left(\eta \right)d\eta ≅\frac{2}{m}\overset{m}{\underset{s=0}{\sum }}\;\overset{m}{\underset{i=0}{\sum }}\frac{\theta_{s}F\left(\eta_{s}\right)}{2i+1}\left[T_{s}^{*}\left(\eta_{2i}\right)-T_{s}^{*}\left(\eta_{2i+2}\right)\right], \end{array}$$

where

(54) $$\begin{array}{c} \theta_{0}=\theta_{m}=\frac{1}{2},\;\;\theta_{s}=1\;\forall s=1,2,\ldots ,m-1,\\ \;\;\eta_{i}=\cos\left[\frac{\left(\pi i\right)}{m}\right]\;\forall i<m,\;\;\eta_{i}=-1\;\forall i>m, \end{array}$$

to approximate the integral (52) as a finite sum of shifted Chebyshev polynomials as follows:

(55) min⁡⁡J =1m∑s=0m ∑i=0mθs2i+1×(12(ηs+1)×[∑r=0Ndηs,r1x(ηr)+∑r=0Ndηs,rαx(ηr)−(12(ηs+1))2]−(α+2)∑n=0N′′anTn∗(ηs))2  ×[Ts∗(η2i)−Ts∗(η2i+2)].

*Step *5. According to the Rayleigh-Ritz method, the critical points of the objective functional (42a) are given by

(56) $$\begin{array}{c} \frac{\partial J}{\partial x\left(t_{1}\right)}=0,\;\frac{\partial J}{\partial x\left(t_{2}\right)}=0,\ldots ,\\ \frac{\partial J}{\partial x\left(t_{N}\right)}=0, \end{array}$$

which leads to a system of nonlinear algebraic equations. Solve this system by Newton's method to obtain *x*(*t*_1_), *x*(*t*_2_),…, *x*(*t*_*N*−1_) and use the boundary conditions to get *x*(*t*_0_),  *x*(*t*_*N*_). Then, the pair (*x*, *u*) which solves the FOCP has the form

(57a) $$\begin{array}{c} x\left(t\right)=\;\frac{2}{N}{\overset{N}{\underset{n=0}{\sum }}}^{\prime \prime }{\overset{N}{\underset{r=0}{\sum }}}^{\prime \prime }x\left(t_{r}\right)T_{n}^{*}\left(t_{r}\right)T_{n}^{*}\left(t\right), \end{array}$$

(57b) u(t)=  x˙(t)+D0Ctαx(t)−t2.

**Figure 5:**
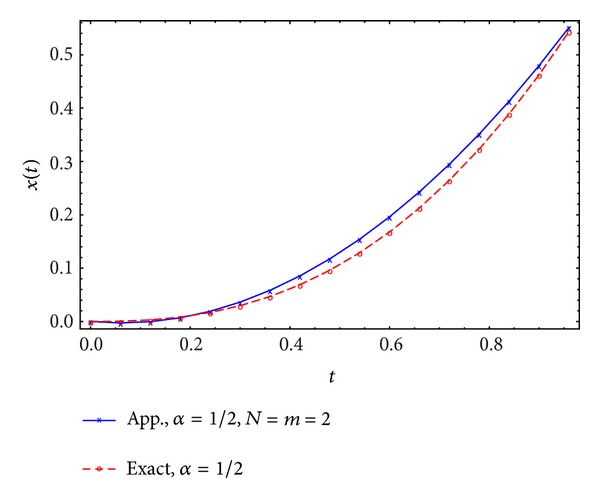
Exact and approximate state.

**Figure 6:**
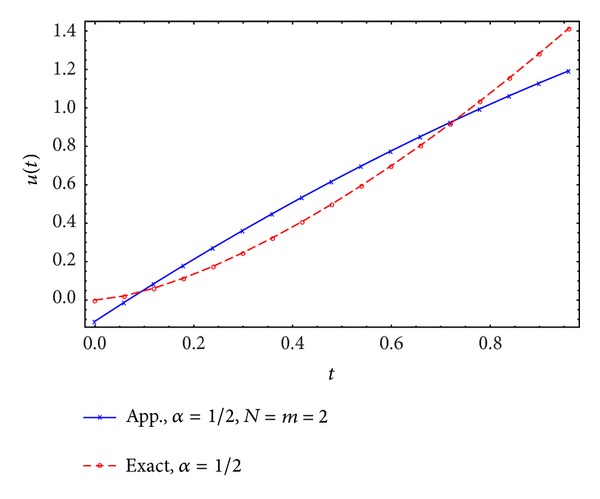
Exact and approximate control.

**Figure 7:**
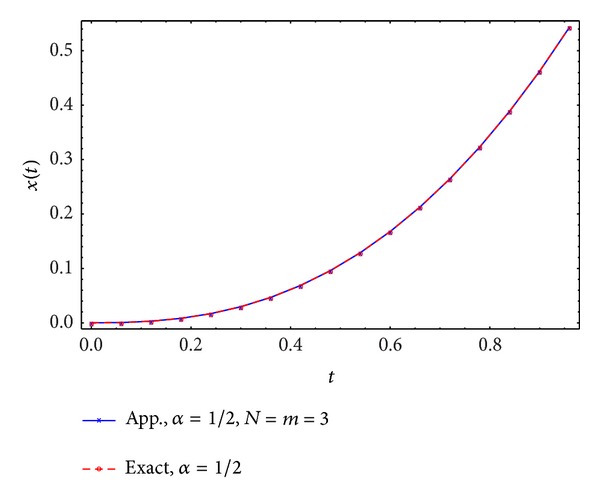
Exact and approximate state.

**Figure 8:**
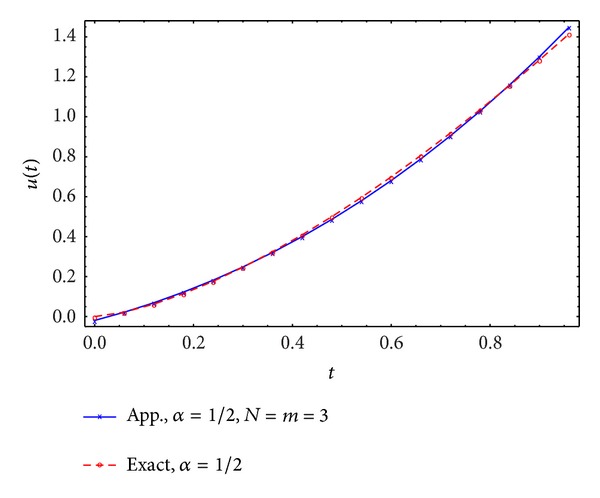
Exact and approximate control.

Figures 5, 6, 7, and 8 display the exact and approximate state *x* and the exact and approximate control *u* for *α* = 1/2 and *N* = *m* = 2,3.

Table 2 contains the maximum errors in the state *x* and in the control *u* for *N* = *m* = 2, *N* = *m* = 3, and *N* = *m* = 5.

A comparison of Tables 1 and 2 reveals that both algorithms yield comparable numerical results which are more accurate than those obtained by the algorithm used in [8].

**Example 2**

We consider the following linear-quadratic optimal control problem:

(58a) $$\begin{array}{c} \min\;J\left(x,u\right)=\overset{1}{\underset{0}{\int }}{\left(u\left(t\right)-x\left(t\right)\right)}^{2}dt, \end{array}$$

subject to the dynamical system

(58b) x˙(t)+D0Ctαx(t)=u(t)−x(t)+6tα+2Γ(α+3)+t3

and the boundary conditions

(58c) $$\begin{array}{c} x\left(0\right)=0,\;x\left(1\right)=\frac{6}{\Gamma \left(\alpha +4\right)}. \end{array}$$

The exact solution is given by

(59) $$\begin{array}{c} \left(\overset{-}{x}\left(t\right),\overset{-}{u}\left(t\right)\right)=\left(\frac{6t^{\alpha +3}}{\Gamma \left(\alpha +4\right)},\frac{6t^{\alpha +3}}{\Gamma \left(\alpha +4\right)}\right). \end{array}$$

We note that, for Example 2, the optimality conditions stated in Theorem 2 are also sufficient (cf.  Remark 3).

Table 3 contains a comparison between the maximum error in the state *x* and in the control *u* for Algorithms A and B.

As opposed to Example 1, in this case, Algorithm A performs substantially better than Algorithm B.

## 6. Conclusions

In this paper, we have presented two algorithms for the numerical solution of a wide class of fractional optimal control problems, one based on the “optimize first, then discretize” approach and the other one on the “discretize first, then optimize” strategy. In both algorithms, the solution is approximated by *N*-term truncated Chebyshev series. Numerical results for two illustrative examples show that the algorithms converge as the number of terms is increased and that the first algorithm is more accurate than the second one.

## Figures and Tables

**Table 1:** Maximum errors in the state *x* and in the control *u* for different values of *N*.

	*N* = 2	*N* = 3	*N* = 5
Max. error in *x *	3.03292*E* − 2	3.4641*E* − 3	2.6415*E* − 4
Max. error in *u *	2.12592*E* − 1	4.1878*E* − 2	7.7493*E* − 3

**Table 2:** Maximum errors in the state *x* and in the control *u* for different values of *N*.

	*N* = *m* = 2	*N* = *m* = 3	*N* = *m* = 5
Max. error in *x *	3.03292*E* − 2	3.4641*E* − 3	2.6416*E* − 4
Max. error in *u *	2.69495*E* − 1	4.8393*E* − 2	8.0532*E* − 3

**Table 3:** 

	Alg. A, *N* = 3	Alg. B, *N* = *m* = 3
max. error in *x *	7.6404*E* − 3	1.1943*E* − 2
max. error in *u *	7.6404*E* − 3	1.6339*E* − 1

	Alg. A, *N* = 5	Alg. B, *N* = *m* = 5

max. error in *x *	7.8604*E* − 5	1.0304*E* − 4
max. error in *u *	7.8604*E* − 5	1.0600*E* − 3
